# Comparative Analysis of Metastatic Thyroid Carcinoma versus Ectopic Thyroid Carcinoma in Lateral Neck Masses without Identifiable Primary Thyroid Carcinoma

**DOI:** 10.3390/jcm13195819

**Published:** 2024-09-29

**Authors:** Hye-kyung Shim, Mi Ra Kim

**Affiliations:** 1 Department of Nuclear Medicine, Haeundae Paik Hospital, Inje University College of Medicine, Busan 48108, Republic of Korea; shimhk80@naver.com; 2 Department of Otorhinolaryngology-Head and Neck Surgery, Haeundae Paik Hospital, Inje University College of Medicine, Busan 48108, Republic of Korea

**Keywords:** metastatic thyroid carcinoma, ectopic thyroid carcinoma, lymph node metastasis in the lateral neck, papillary thyroid carcinoma

## Abstract

**Background/Objectives:** Thyroid carcinoma, presenting as a lateral neck mass without an identifiable primary tumor within the thyroid, poses a diagnostic challenge. This comparative analysis aimed to explore the differences between metastatic thyroid carcinoma and ectopic thyroid carcinoma, as both present with a lateral neck mass without evidence of primary thyroid carcinoma. **Methods:** Searches were conducted for studies on thyroid carcinoma in the lateral neck without evidence of primary thyroid carcinoma. A total of 39 patients were identified from 32 reported studies. **Results:** Metastatic and ectopic thyroid carcinomas were found in 11 and 28 patients, respectively. Metastatic thyroid carcinoma is characterized by evidence of spontaneous primary tumor regression within the thyroid and commonly associated with multiple lymph node metastases in central and lateral neck compartments. Ectopic thyroid carcinoma is more commonly diagnosed in younger patients and is frequently identified in branchial cleft cysts. The coexistence of normal thyroid tissue adjacent to the ectopic thyroid carcinoma was confirmed, and patients with ectopic thyroid carcinoma exhibited significantly higher rates of second-stage thyroidectomy or neck dissection. When complete surgical excision was considered adequate, excision alone was chosen for patients with ectopic thyroid carcinoma. **Conclusions:** Identifying these differences is valuable for the differential diagnosis and development of treatment strategies for metastatic and ectopic thyroid carcinomas in lateral neck masses without evidence of primary thyroid tumor.

## 1. Introduction

The majority of metastatic thyroid carcinomas without a detectable primary tumor within the thyroid on preoperative evaluation can be identified as primary thyroid carcinomas after total thyroidectomy [1,2]. With advancements in diagnostic techniques and the discovery of new promising diagnostic biomarkers [3], the preoperative detection of primary thyroid carcinoma and lymph node metastasis has considerably improved. A metastatic lateral neck mass could be an initial presentation of papillary thyroid carcinoma (PTC) because of the significant potential for lymphatic metastasis regardless of the primary tumor size [1]. A comprehensive evaluation of the entire neck and thyroid is essential when thyroid carcinoma is diagnosed in the lateral neck.

An ectopic thyroid, defined as the presence of thyroid tissue outside the original location, results from embryonic developmental abnormalities [4]. Ectopic thyroids in the lateral neck are rare (1–3% of all ectopic thyroids) [5,6]. Ectopic thyroids can develop into malignancies, with PTC being the most commonly identified malignancy [4,7]. Ectopic thyroid carcinoma in the lateral neck is challenging to diagnose because of its extreme rarity and clinical manifestations that resemble metastatic thyroid carcinoma or a benign mass [7].

For the management of thyroid carcinoma in the lateral neck, previous studies suggest total thyroidectomy with neck dissection, followed by adjuvant radioactive I-131 therapy tailored to individual risk stratification [5,6,7,8,9,10,11,12,13]. If primary thyroid carcinoma is undetected after a histopathological evaluation of the thyroid, diagnosis becomes challenging. A few studies have reported thyroid carcinoma presenting as a lateral neck mass without a detectable primary tumor through a radiological and histopathological examination of the thyroid [1,2,8,14,15,16,17]. Its pathogenesis, clinical manifestations, diagnosis, treatment, and prognosis remain undefined. This study aimed to compare and highlight the distinguishing characteristics of metastatic thyroid carcinoma (metastatic TC), presenting as a lateral neck mass and thyroid carcinoma originating from ectopic thyroid tissue (ectopic TC) in the lateral neck, both without evidence of a primary thyroid tumor.

## 2. Methods

### 2.1. Search Criteria

Searches were conducted for English-language studies published from 1980 to 2023 to identify research on thyroid carcinomas diagnosed in the lateral neck with undetectable primary thyroid tumors. The databases used for this search included PubMed, Ovid Medline, and Embase. The searches were performed using the following keywords: ‘‘lymph node metastasis in lateral neck without primary thyroid carcinoma/cancer/tumor”, ‘‘thyroid carcinoma/cancer in lateral neck mass without primary thyroid carcinoma/cancer/tumor”, “occult thyroid carcinoma/cancer”, “lateral aberrant thyroid carcinoma/cancer”, and “ectopic thyroid carcinoma/cancer and lateral neck mass”. However, studies that identified metastatic lymph nodes in the lateral neck and primary thyroid carcinoma were excluded from this analysis. Furthermore, this study excluded cases of lymph node metastasis in the lateral neck that were associated with thyroid carcinoma diagnosed in the anterior neck, such as those with thyroglossal duct cysts or lingual thyroid. A comparative analysis was performed, and 39 patients were identified from 32 studies of thyroid carcinoma presenting as a lateral neck mass without an identifiable primary thyroid tumor.

### 2.2. Representative Case and Literature Review

A 31-year-old man presented with a solitary lateral neck cystic mass confirmed as PTC. However, after total thyroidectomy and neck dissection, comprehensive radiological and histopathological examinations showed no primary thyroid tumor. The Institutional Review Board approved this study (Approval Code: HPIRB 2020-09-010 and Approval Date: 24 September 2020).

### 2.3. Diagnosis, Treatments, and Prognosis

Radiological studies, such as ultrasonography (US), computed tomography (CT), and US-guided fine-needle aspiration and biopsy (US-FNAB), are primarily used for diagnosis. Washout thyroglobulin (Tg) in the FNAB sample was measured in one reported case [18] and in the present patient. F-18 fluorodeoxyglucose (FDG) positron emission tomography/CT (PET/CT) is a useful method for evaluating primary tumors, staging, assessing recurrence and therapeutic response, and predicting prognosis in thyroid cancer. The clinical manifestations, radiological and histopathological findings, diagnostic processes, treatments of the thyroid and neck, and prognoses were summarized. The results of the comparative analysis, elucidating the differences between metastatic TC and ectopic TC, are documented in Tables 1 and 2.

### 2.4. Statistical Analyses

Statistical analyses were performed using the IBM SPSS Statistics software (ver. 25.0) (SPSS Inc., Chicago, IL, USA). This comparative analysis included 39 patients from 32 studies. The demographic and clinical characteristics, results of radiological and histopathological examinations, treatment strategies, and prognosis of the metastatic TC and ectopic TC groups were compared using the *t*-test, Mann‒Whitney U test, chi-square test, and Fisher’s exact test, as appropriate. All data are presented as the means ± standard deviation. A *p*-value < 0.05 was considered statistically significant.

## 3. Results

### 3.1. Representative Case of PTC in Solitary Lateral Neck Cystic Mass without Identifiable Primary Carcinoma in Thyroid

**Figure 1:**
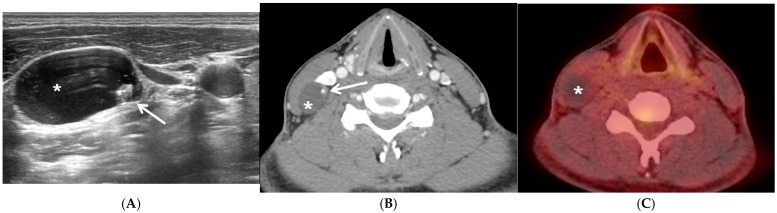
Representative case of papillary thyroid carcinoma in solitary lateral neck cystic mass without identifiable primary carcinoma within the thyroid. Preoperative ultrasonography (US) (**A**) and computed tomography (CT) (**B**) showing a 25 mm × 16 mm-sized lobular, well-defined, cystic mass (asterisk) containing a solid and hyperechoic lesion with punctate calcification (white arrow) in the right neck at level III (**A**,**B**) without other enlarged lymph nodes on either side of the neck and normal findings in the thyroid. The cystic lateral neck mass in the right neck exhibited no obvious F-18 fluorodeoxyglucose (FDG) uptake or any other suspicious F-18 FDG-avid lesions documented on positron emission tomography/CT (PET/CT) (**C**).

A 31-year-old man presented with a soft, painless, non-tender mass in the right lateral neck that had persisted for 1 month. US and contrast-enhanced CT revealed a cystic mass on the right neck at level III (Figure 1A,B). No abnormal lesions were observed in the thyroid on US or CT. The FNAB result was consistent with PTC, and the increased washout Tg levels (>500 ng/mL) facilitated diagnosis. F-18 FDG PET/CT was performed to exclude other possible malignancies that could metastasize to the lateral neck. The mass exhibited no obvious F-18 FDG uptake or other hypermetabolic lesions on F-18 FDG PET/CT (Figure 1C). The preoperative diagnosis was a presumed metastatic thyroid carcinoma in the lateral neck. After excising the cystic mass, the PTC was identified using frozen section analysis. The patient underwent total thyroidectomy, along with a comprehensive neck dissection of the central (both level VI) and lateral neck compartments (right, levels II–V). The patient was managed with levothyroxine replacement therapy.

The histopathological examination revealed a unifocal 8 mm × 7 mm PTC in the lymphoid tissue of only one lymph node out of the 71 resected lymph nodes. The lesion was resected with a clear resection margin and was associated with extranodal extension. There were no coexisting normal thyroid tissues adjacent to the carcinoma. The primary thyroid carcinoma was not detected despite a comprehensive review of histopathological examinations of serial thin sections and immunohistochemical (IHC) studies of total thyroidectomy specimens. Moreover, no lesions showed spontaneous regression. Mutated BRAF V600E was detected by IHC analysis. This patient was reviewed in a multidisciplinary discussion, which suggested ectopic TC rather than metastatic TC. However, the possibility of metastatic TC could not be completely excluded. Upon diagnosis with metastatic TC, the patient was staged as pT0N1bM0, stage I [19], and he had an intermediate-to-high risk of recurrence [9]. Adjuvant radioactive iodine ablation therapy with 3700 MBq iodine-131 (I-131) was administered 2 months later. The patient was in good condition, with no clinical, biochemical, or structural evidence of recurrence after 50 months of follow-up.

### 3.2. Comparative Analysis of Demographic and Clinical Characteristics between Metastatic TC and Ectopic TC

Thyroid carcinoma was confirmed in a lateral neck mass without a detectable primary tumor in 39 patients and the present patient. The demographic and clinical characteristics of patients with metastatic TC and ectopic TC are presented in Table 1. Among the 39 reported patients, 11 were diagnosed with metastatic TC [1,2,11,14,15,16,17], whereas 28 were diagnosed with ectopic TC. The mean age of patients with ectopic TC was lower compared to those with metastatic TC, although this difference did not achieve statistical significance (36.0 ± 12.4 years vs. 50.4 ± 20.7 years, *p* = 0.054). No statistically significant differences were observed in the sex of the patients or the size of the lateral neck mass between the two groups. PTC was the most commonly diagnosed thyroid carcinoma in lateral neck masses (89.7%, 35/39). There was one case each of follicular [20] and medullary thyroid carcinomas [21] in ectopic TC and one case each of poorly differentiated [1] and anaplastic thyroid carcinomas [1] in metastatic TC. Ectopic TC was most commonly identified within branchial cleft cysts (BCCs) in the lateral neck (67.9%, 19/28) [13,18,22,23,24,25,26,27,28,29,30,31,32,33,34,35]. It presented as thyroid inclusions within the lymph nodes in five patients [5,8,36,37,38] and as a sequestered mass in four patients [12,20,21,39]. Normal thyroid tissue adjacent to the thyroid carcinoma was identified in 10 patients with ectopic TC (35.7%, 10/28) [12,18,20,28,29,30,31,32,33,38]; however, this feature was not observed in 6 patients (21.4%, 6/28) due to the tumor-induced effacement of the normal tissue [13,34,35,36]. Imaging studies, including US and CT of the thyroid and neck, were performed as a preoperative evaluation of the lateral neck cystic mass in most cases. A total of five patients underwent PET/CT, including two patients with metastatic TC [15,17] and three patients with ectopic TC [25,34,36]. None of the five patients exhibited increased FDG uptake in the thyroid, nor were there any suspected distant metastases. Additionally, 10 patients underwent whole-body iodine scans, including 1 patient with metastatic TC [17] and 9 patients with ectopic TC [5,8,12,27,30,33,36,38,39], and no distant metastases were detected in any of these patients. Owing to the challenges in the preoperative differential diagnosis between ectopic TC and benign cysts, seven patients initially diagnosed with benign cysts underwent surgical excision, which revealed thyroid carcinoma within the BCC through histopathological analysis, emphasizing the need for comprehensive evaluations of the thyroid and neck [13,25,26,27,28,29,30].

**Table 1:** Differences in the demographic and clinical characteristics of patients with metastatic versus ectopic thyroid carcinoma in the lateral neck mass without an identifiable primary thyroid tumor.

	Metastatic TC (*n* = 11)	Ectopic TC (*n* = 28)	*p*-Value
Age (years; range)	50.4 ± 20.7 (17–76)	36.0 ± 12.4 (15–75)	0.054
Sex (Male/Female) (*n*)	47	13/15	0.725
Lateral neck mass size (mm; range)	34.7 ± 16.5 (15–68)	44.7 ± 22.6 (20–100)	0.137
Histopathological results of lateral neck mass (*n*)	Metastatic thyroid carcinoma (*n* = 11)	Thyroid carcinoma within BCC (*n* = 19) LN (*n* = 5) Sequestered mass (*n* = 4)	
Histopathological subtype of thyroid carcinoma (*n*); PTC/others	9/2	26/2	0.562
Thyroid evaluation (*n*); Preop/After excision of mass/NA	7/13	17/7/4	0.648

Abbreviations: *n*, number of patients; BCC, branchial cleft cyst; ectopic TC, thyroid carcinoma originating from the ectopic thyroid tissue in the lateral neck, without evidence of a primary thyroid tumor; LN, lymph node; metastatic TC, metastatic thyroid carcinoma, which presents as a lateral neck mass without evidence of a primary thyroid tumor; NA, not available; Preop, preoperative; PTC, papillary thyroid carcinoma; means ± standard deviation.

### 3.3. Comparative Analysis of Treatment Strategies, Histopathological Results, and Prognosis of Metastatic TC versus Ectopic TC

The treatment of the thyroid (*p* = 0.009) and neck (*p* = 0.005) differed significantly between the two groups (Table 2). In the metastatic TC group, one patient, who did not undergo thyroidectomy with neck dissection, was diagnosed with plausible metastatic TC [11], while total thyroidectomy was performed on 10 patients. Spontaneous regression in the thyroid was confirmed in four patients [1,14,17]. In the remaining six patients, no specific findings were noted in the thyroid; however, they were classified as having metastatic TC based on a comprehensive review of all the results. Patients with metastatic TC exhibited a significantly higher incidence of neck dissection (*p* = 0.005) which was accompanied by multiple lymph node metastases in the lateral and central neck compartments (*p* = 0.032). Two patients diagnosed with metastatic TC did not undergo neck treatment: one, with plausible metastatic TC, underwent only the excision of the lateral neck mass [11], whereas the other underwent total thyroidectomy without neck dissection [16]. For patients with preoperative metastatic TC, total thyroidectomy with neck dissection was predominantly performed, and no additional second-stage surgeries were observed.

**Table 2:** Differences in the treatment, histopathological results, and prognosis of patients with metastatic versus ectopic thyroid carcinoma in the lateral neck mass without an identifiable primary thyroid tumor.

	Metastatic TC (*n* = 11)	Ectopic TC (*n* = 28)	*p*-Value
Thyroid treatment (*n*); TT/HT/2nd TT/2nd HT/not conducted	10/0/0/0/1	9/0/13/1/5	0.009 *
Thyroid treatment (*n*); Single/Second-stage	10/0	9/14	0.001 *
Neck treatment (*n*); Not conducted/ND/2nd ND/NA	2/9/0/0	10/6/9/3	0.005 *
Neck treatment (*n*); Single/Second-stage	9/0	6/9	0.002 *
Accompanied by metastatic LN (*n*); No/Single/Multiple/NA	1/2/6/0	8/2/3/2	0.032 *
RIT (*n*); Yes/No/NA	4/1/6	9/8/11	0.637
Prognosis; NED/Rec/NA (*n*) NED (duration (months))	6/2/3 116 ± 77.9 (24–204)	18/1/9 40.9 ± 25.9 (12–96)	0.201

Abbreviations: *n*, number of patients; 2nd, second stage; ectopic TC, thyroid carcinoma originating from the ectopic thyroid tissue in the lateral neck, without evidence of a primary thyroid tumor; HT, hemithyroidectomy; LN, lymph node; metastatic TC, metastatic thyroid carcinoma, which presents as a lateral neck mass without evidence of a primary thyroid tumor; NA, not available; ND, neck dissection; NED, no evidence of disease; Rec, recurrence; RIT, radioactive I-131 therapy; TT, total thyroidectomy; means ± standard deviation; * A *p*-value < 0.05 was taken to indicate statistical significance.

Conversely, in the ectopic TC group, total thyroidectomy was performed on 9 patients, second-stage total thyroidectomy on 13 patients, and second-stage hemithyroidectomy on 1 patient [23]. Thyroid carcinoma was diagnosed following the excision of the lateral neck mass, initially presumed to be benign, necessitating a second-stage thyroidectomy and neck dissection. Patients with ectopic TC showed significantly higher rates of second-stage thyroidectomy (*p* = 0.001) and second-stage neck dissection (*p* = 0.002) after excision compared to those with metastatic TC. Ten patients diagnosed with ectopic TC did not undergo neck treatment: five patients with ectopic TC within a BCC were closely observed following lateral neck mass excision [22,25,28,35], whereas a second-stage total thyroidectomy without neck dissection was performed in four patients with ectopic TC within a BCC [27,29,33,35] and in one patient with ectopic TC within a lymph node in the lateral neck [8]. The prognosis of ectopic TC appears to be relatively favorable; however, the difference was not statistically significant compared to metastatic TC (*p* = 0.201). The limited follow-up data complicate the ability to predict prognosis. Given the rarity of these cases, a more extended follow-up period would provide better insights into recurrence rates and overall survival.

## 4. Discussion

The commonly observed characteristics of thyroid carcinoma within a lateral neck mass, without a detectable primary thyroid carcinoma in reported studies, including case reports, were analyzed to differentiate metastatic TC and ectopic TC. The following findings are indicative of metastatic TC. First, evidence of spontaneous primary tumor regression was identified within the thyroid [1,14,17]. Second, metastatic TC is typically accompanied by the multifocal involvement of the lymph nodes of the lateral and central neck compartments [12,30,40]. Conversely, the following findings are suggestive of ectopic TC. First, the presence of normal thyroid tissue adjacent to the thyroid carcinoma within the lateral neck mass was identified [4,6,12,17,18,20,28,29,30,31,32,33,34,38,41,42,43,44]. However, diagnosis can be challenging if normal thyroid tissues are effaced by tumor growth or cystic degeneration [13,29,34,35,36]. Second, concurrent metastatic lymph nodes were usually not identified, until the disease progressed to advanced metastatic stages. Moreover, metastases to the lymph nodes in the central neck compartment are rare [45,46,47]. Third, ectopic TC was more frequently diagnosed in younger age groups. Although the commonly observed features in metastatic TC and ectopic TC are comparable, diagnostic challenges remain, as in the present case. This patient presented with no coexisting normal thyroid tissues adjacent to the carcinoma, no lesions indicative of spontaneous regression, and no other concurrent metastatic lymph nodes. A multidisciplinary review suggested that this case was more suggestive of ectopic TC rather than metastatic TC. An appropriate diagnosis and treatment strategy may be developed through multidisciplinary discussions incorporating clinical, radiological, and histopathological findings.

Although the pathogenesis of undetectable primary thyroid carcinoma in metastatic TC remains unclear, two possibilities have been proposed [1,5,14,17,48]. The first possibility is that the primary thyroid carcinoma was overlooked [2,10,48], emphasizing the need for a comprehensive histopathological examination of the thyroid following thyroidectomy, using serial thin sections and additional IHC staining [48,49]. Another possibility is the spontaneous regression of primary thyroid carcinomas [1,5,14,17,48]. Spontaneous tumor regression is defined as the diminution of a malignant neoplasm as a consequence of the host immune response, which plays an important role despite its unclear pathogenesis [1,5]. Intra-tumoral hyalinization and fibrotic changes may suggest tumor regression in the thyroid [1,14,17]. Additionally, the possibility of ectopic TC should be considered [29,50].

The utility of F-18 FDG PET/CT in carcinoma of unknown primary origin is already well established. However, in cases of metastatic TC, diagnosis may be challenging when the primary thyroid cancer is small or well differentiated. F-18 FDG uptake reflects the degree of differentiation, thus serving as a prognostic factor. Radioactive iodine is commonly used for the diagnosis and therapeutic management of thyroid disorders. When radioactive iodine is administered for therapeutic or diagnostic purposes in metastatic TC, primary or other metastatic lesions may be suspected on a whole-body scan, and specificity can be enhanced by combining it with CT imaging. Consequently, F-18 FDG PET/CT and whole-body radioactive iodine scans complement each other. Moreover, F-18 FDG PET/CT and whole-body radioactive iodine scans are valuable diagnostic modalities in the evaluation of ectopic TC [51]. There have been reports of ectopic thyroid cancer demonstrating a max SUV of 5.5–25 g/mL on F-18 FDG PET/CT, despite the limited number of patients [51]. In ectopic TC, using radioactive iodine scans in follow-up examinations offers the advantage of identifying the location of benign thyroid remnants or metastatic lesions. In the representative patient as well, no significant F-18 FDG uptake was observed in the thyroid, and PET/CT confirmed the absence of distant metastases. Additionally, no lesions suspicious for metastasis were found on the post-radioactive iodine treatment scan. In addition to F-18 FDG, PET-CT using F-18 DOPA, Ga-68 DOTATATE, and Ga-68 FAPI are also valuable diagnostic tools, though their widespread use is limited.

Molecular and genetic markers for distinguishing between metastatic TC and ectopic TC have remained undefined. The presence of a BRAF mutation and overexpression of the Cyclin D1 protein have been predominantly observed in lymph node metastasis, suggesting that these factors may play an important role in metastasis [1,16]. In addition, liquid biopsy, a less invasive diagnostic method that analyzes circulating tumor components, including cell-free DNA, circulating tumor cells, exosomes, and microRNAs, to detect genetic mutations, tumor markers, and monitor treatment response, is being actively investigated [3]. It is essential to further investigate the role of molecular and genetic markers in distinguishing metastatic TC from ectopic TC.

There is controversy regarding the extent of surgical treatment due to the difficulty in preoperative differential diagnosis between metastatic TC and ectopic TC. In patients diagnosed with presumed metastatic TC, the thyroid and neck were treated, which likely accounts for the rarity of second-stage surgery. The incidence of second-stage thyroidectomy and neck dissection following the excision of a lateral neck mass was statistically significantly higher in patients with ectopic TC, due to challenges in preoperatively distinguishing it from benign cystic diseases. However, when radiological studies did not reveal any abnormal findings in the thyroid or neck, excision alone was chosen [7,8,12,13,20,22,25,27,28,29,33,35]. In 10 patients diagnosed with ectopic TC who did not undergo neck treatment, no recurrence was observed in 8 patients over a follow-up period of 36 to 96 months [8,22,25,29,33,35], except for 2 patients whose prognosis could not be assessed [27,28]. Therefore, in patients diagnosed with ectopic TC, when complete surgical excision was deemed sufficient, close observation might be considered as an alternative to proceeding with thyroidectomy or neck dissection [7,8,12,13,20,22,25,27,28,29,33,35]. Thyroid cancer with lymph node metastasis is known to have a locoregional recurrence rate of approximately 30% after treatment [52] and is associated with worse overall survival [53]. Metastatic TC represents a more advanced stage of cancer compared to ectopic TC, leading to greater psychological distress for patients due to the complexity of treatment and increased risk of recurrence or metastasis, which can negatively affect the quality of life. Therefore, identifying the impact of metastatic TC and ectopic TC on treatment and prognosis, as well as psychological perspective, is important.

## 5. Conclusions

This research compares metastatic TC and ectopic TC based on reported studies. Metastatic TC is characterized by the presence of a lesion suspicious for the tumor regression of the primary thyroid carcinoma and frequently accompanied by multiple metastatic lymph nodes in the lateral and central neck compartments. Conversely, ectopic TC is often observed to be coexisting with normal thyroid tissue adjacent to the carcinoma and shows significantly higher rates of second-stage thyroidectomy or neck dissection. When ectopic TC presenting as a solitary lateral neck mass is diagnosed preoperatively, complete surgical removal followed by regular clinical and radiological studies can be carefully considered a therapeutic option for well-selected patients. Further systematic studies with larger patient populations and extended follow-up data are necessary to more definitively elucidate these differences, which are crucial for developing treatment strategies and improving the understanding of long-term outcomes and recurrence risks.

## Data Availability

All data generated or analyzed during this study are included in this published article. Further inquiries should be directed to the corresponding author.

## Abbreviations

Ectopic TC: thyroid carcinoma originating from the ectopic thyroid tissue in the lateral neck without evidence of a primary thyroid tumor. Metastatic TC: metastatic thyroid carcinoma, which presents as a lateral neck mass without evidence of a primary thyroid tumor.
